# Impact of Delivery Method on Serum Cortisol Levels and Neonatal Outcomes in Canine Cesarean Sections

**DOI:** 10.3390/ani15121739

**Published:** 2025-06-12

**Authors:** Renatha Almeida de Araújo, João Domingos Rocha-Júnior, Jaqueline Tamara Bonavina, Melissa de Oliveira Bianchini, Samara Beretta, Daniella Jorge Coutinho Armani, Marina Vilela Estevam, Gilson Hélio Toniollo, Henry David Mogollón García, Eunice Oba, Maricy Apparício

**Affiliations:** 1 Department of Pathology, Reproduction and One Health, School of Agricultural and Veterinary Studies, São Paulo State University, FCAV/UNESP, Via de Acesso Prof. Paulo D. Castellane s/n., Jaboticabal 14884-900, SP, Brazil; renatha.araujo@unesp.br (R.A.d.A.); j.rocha@unesp.br (J.D.R.-J.); melissa.bianchini@unesp.br (M.d.O.B.); samara.beretta@unesp.br (S.B.); marina.estevam@unesp.br (M.V.E.); gilson.toniollo@unesp.br (G.H.T.); 2 Department of Veterinary Surgery and Animal Reproduction, School of Veterinary Medicine and Animal Science, São Paulo State University (FMVZ/UNESP), Rua Professor Doutor Walter Mauricio Correa, s/n., Botucatu 18618-681, SP, Brazil; jaqueline.bonavina@unesp.br (J.T.B.); djc.armani@unesp.br (D.J.C.A.); mogollon.garcia@unesp.br (H.D.M.G.); eunice.oba@unesp.br (E.O.)

**Keywords:** Apgar score, distress, dystocia, neonatal viability, progesterone

## Abstract

In this study, thirty female dogs, aged one to five years and varying in weight, in the last week of gestation were evaluated. The animals were divided into two groups: GC, which comprised twenty-two bitches undergoing elective cesarean section, and GD, which consisted of eight dogs requiring therapeutic cesarean section as a treatment to dystocia. We found that cortisol levels in the amniotic fluid were significantly higher in pups delivered via elective cesareans (mean: 9.86 ng/mL) compared to those from therapeutic c-sections (mean: 4.11 ng/mL). This observation contrasted with previous studies that reported lower cortisol levels in elective procedures, suggesting complexities in the physiological responses to different delivery methods that warrant further investigation. Notably, our study observed no significant association between amniotic fluid meconium presence and other distress markers, indicating that meconium may be more closely associated with fetal maturation rather than distress (*p* > 0.05). Neonatal viability (Apgar score) revealed that 92.86% of the neonates from elective procedures demonstrated no distress shortly after delivery, contrasting with 56.25% in therapeutic c-section. Fetal distress can be a direct consequence of dystocia caused by various stressors, such as pain and hypoxia. These factors can impair the fetus’ ability to adapt to extrauterine life, often leading to lower Apgar scores. Notably, neonatal weight was directly related to fetal cortisol levels, while no significant associations were noted between the litter size or birth order and cortisol concentrations, irrespective of the delivery type. These findings underscore the need for ongoing investigation into the relationships between cesarean delivery types, maternal and neonatal stress markers, and resultant health outcomes, aiming to enhance care strategies for expectant canine mothers and their puppies.

## Simple Summary

This study evaluated pregnant dogs undergoing cesarean sections, comparing cortisol levels in amniotic fluid between elective and therapeutic procedures. Higher cortisol was found in pups from elective cesareans compared to therapeutic c-sections. No link was observed between meconium presence and distress markers. Findings highlighted complex physiological responses and the importance of maternal and neonatal stress assessment to improve care strategies.

## 1. Introduction

The birth process marks the end of gestation, characterized by significant neuroendocrine adaptations and modifications of the reproductive system to facilitate neonatal expulsion [1]. Understanding the physiological processes of birth is critical for identifying causes and making informed decisions about the management of potential birth complications [2]. Dystocia, a common emergency in veterinary medicine, poses higher risks of maternal and fetal mortality [3]. Its underlying causes can involve both the dam and the offspring, including congenital malformations, pelvic trauma, neoplasia or abscess, vaginal stricture, uterine torsion, uterine or vaginal prolapse, fetal anatomical abnormalities, malposition, or disproportion [2]. Breed predispositions for canine dystocia have also been observed, such as cephalo-pelvic disproportion in brachycephalic breeds, which can lead to obstructive dystocia [3].

Uterine inertia, characterized by the absence of uterine contractions, is the most common cause of maternal dystocia. It can be primary, when the contractions do not initiate, or secondary, when they cease due to myometrial exhaustion after a period of contractions [2]. The number of puppies in the litter is a significant risk factor; small litters (fewer than three pups) are often insufficient to produce enough cortisol to trigger labor, while very large litters may cause excessive uterine distension impairing contractions [3]. In cases of primary inertia, when contractions fail to begin, inadequate monitoring can lead to both fetal and maternal mortality.

In canine reproduction, accurately monitoring pregnant bitches is crucial for the successful timing of cesarean sections. Determining the ideal delivery time is complicated by natural variations in gestation lengths. The reliance solely on mating dates for estimating delivery time is insufficient, necessitating a combination of multiple diagnostic methods to improve prediction accuracy [4]. Given the short gestation period in dogs, each day is critical for fetal development, significantly affecting neonatal morbidity and mortality. Therefore, the accurate timing of cesarean interventions is pivotal to ensure that the fetuses are born at an optimal gestational age [5].

Cortisol plays a vital role in signaling parturition by regulating key physiological mechanisms. Towards the end of gestation, the increased production of adrenocorticotropic hormone (ACTH) elevates fetal cortisol levels, which initiates the conversion of progesterone to estrogen, resulting in a significant drop in progesterone levels from 4 to 10 ng/mL during gestation to less than 2 ng/mL before birth [6]. Elevated cortisol levels highlight its critical role in the onset of labor, as demonstrated by increased cortisol production during birth, which remains high 12 h after labor begins and decreases 36 h later [6,7].

This study focused on evaluating and comparing cortisol levels in maternal blood and amniotic fluid obtained during therapeutic (dystocia) and elective cesarean sections. We aimed to investigate the relationship between these cortisol levels and neonatal viability, while also assessing correlations with factors such as neonatal birth order, birth weight, and the presence of meconium. Ultimately, this study sought to determine whether cortisol levels can serve as potential indicators of maternal and neonatal outcomes in cesarean procedures.

## 2. Materials and Methods

This study was approved by the Ethical Committee of the Institution (Protocol 011302/19).

### 2.1. Animals

Thirty female dogs, aged between two and five years with varying weights, in the final third of gestation were used. Among these, 22 underwent an elective cesarean section (GC group)—consisting of 16 English Bulldogs, 4 French Bulldogs, and 2 Chow Chows (with a total of 94 puppies)—while 8 animals experienced dystocia (GD group)—including 1 Shih-Tzu, 1 English Bulldog, 3 American Bullies, 1 Border Collie, 1 pinscher and 1 mixed breed, resulting in a total of 20 puppies.

The female dogs in the elective cesarean group (GC) were monitored by the Animal and Reproduction service (SORA) at the Veterinary Hospital. Gestational timing was determined considering the mating date (between 58 to 63 days), alongside fetal maturation variables assessed through ultrasonographic examination, as described by [8], and radiography. The appropriate time for cesarean section was established based on these evaluations, including the observation of a drop in rectal temperature (0.5 to 1 °C), monitored every six hours from the 56th day post-mating/insemination. Delivery was estimated to occur approximately 24 h after the temperature reached 37.5 °C. Normal parturition typically follows a sudden decline in progesterone levels from 4 to 10 ng/mL during pregnancy to less than 2 ng/mL 1–2 d before whelping. This progesterone cutoff value was used for evaluation, as recommended by [9]. Serum progesterone levels were measured using an electrochemiluminescence immunoassay.

### 2.2. Selection Criteria

Pregnant dogs exhibiting the signs of dystocia were diagnosed to determine whether the condition had a maternal or fetal origin, with only those experiencing maternal-origin dystocia included in GD group. The duration of labor onset was also a limiting factor; thus, only cases of dystocia with a progression of no more than 6 h from the second stage of labor were included in this study.

Dystocic deliveries were classified by the presence of one or more of the following characteristics: the absence of abdominal contractions, more than 30 min of intense contraction without fetal expulsion, clear signs of the expulsion phase lasting over two hours without fetal expulsion, evident maternal distress, rupture of the fetal membranes without fetal expulsion, the presence of greenish secretion without fetal expulsion, and fetal distress (characterized by a persistent heartbeat equal of 170 bpm or lower).

### 2.3. Surgical Procedure and Neonatal Management

Before the surgical procedure, all owners signed an informed consent form (ICF) after being informed about the study procedures and the use of results for scientific dissemination. The female dogs underwent clinical and laboratory examinations, including a complete blood test, serum creatinine and ALT levels, to determine their health status and ensure the safety of subsequent procedures.

Regardless of whether the surgery was elective or therapeutic, anesthetic induction was performed using propofol at a dose of 2 mg/kg for orotracheal intubation, and maintenance was achieved using isoflurane vaporization. Additionally, a regional block with 2% lidocaine at a dose of 2 mg/kg was performed via the epidural route.

Neonatal care was provided by a trained team. Resuscitation involved clearing the airway and relieving obstructions, stimulating breathing through chest friction with compresses, and careful drying to prevent hypothermia. Neonatal vitality was assessed using the modified Apgar score [10] at M0 (immediately after cardiopulmonary resuscitation) and M5 (5 min after). All puppies were evaluated for possible congenital abnormalities, and their gender and weight were recorded.

### 2.4. Sample Collection

Amniotic fluid was collected by puncturing the amniotic sac before it ruptured. The samples were centrifuged at 2500× *g* for 10 min and the supernatant was aliquoted and stored in an Eppendorf tube at −20 °C. Samples containing meconium or originating from neonates with any anomalies or malformations were identified for later correlation with measured cortisol values.

Maternal blood samples were collected via jugular venipuncture immediately before the surgical procedure. These samples were centrifuged at 2500× *g* for 10 min, and the serum was aliquoted and stored in Eppendorf tubes at −20 °C for a maximum of 3 months.

### 2.5. Analysis of Fetal Fluids and Maternal Blood

Amniotic fluid samples and blood samples were analyzed for cortisol levels using the Beckman Coulter© Cortisol RIA Kit for radioimmunoassay (cortisol kit, catalog number IM 1847, Prague, Czech Republic). The inter- and intra-assay coefficients of variation were 4.5% and 3.6%, respectively. These samples were sent to the Endocrinology Laboratory at the School of Veterinary Medicine and Animal Science—FMVZ, Unesp, Botucatu—SP, where cortisol levels were determined with the Perkin Elmer 1470 automatic device, following the manufacturer’s instructions for the commercial solid-phase kit. The same technique was used by Bolis et al. (2017) [11].

Maternal blood samples were analyzed for progesterone levels using COBAS^®^ e411 Roche Diagnostics International Ltd., Rotkreuz, Switzerland (catalog number 07092539190), following the manufacturer’s instructions.

### 2.6. Statistical Analysis

The assumption of normality was assessed using the Shapiro–Wilk test. When necessary, logarithm transformation was performed. The parametric approach included the unpaired *t*-test and ANOVA for the P4, cortisol (mother vs. litter), and cortisol (dam vs. litter) variables, respectively. Results are shown as mean ± standard error of the mean (SEM). Cortisol of the dams (GC and GD) were analyzed using a descriptive approach. The remaining variables were analyzed with the non-parametric Mann–Whitney test. The results are shown as median (Q1 and Q3). A significant difference was considered when *p* < 0.05.

Spearman correlation between cortisol and pup weight was performed. The results are presented as a correlation coefficient. Strong, moderate, and weak correlation coefficients were considered when r > 0.6; 0.6 ≤ r ≥ 0.4, and r < 0.4, respectively. Statistical difference was estimated when *p* < 0.05.

The SAS (https://welcome.oda.sas.com/, accessed on 20 March 2025) and GraphPad Prism (Version 9.3.0) packages were used to perform the statistical analysis.

## 3. Results

### 3.1. General Information

All the bitches in the dystocia group exhibited uterine inertia. The litter size in GC ranged from one to nine, with a mean of 4.3 ± 2.2 SD, while the birth weight varied from 148 to 554 g (mean ± SD: 315.2 ± 82.2 g). In group GD, the litter size ranged from one to four (2.5 ± 1.3), and the birth weight ranged from 78 to 376 g (mean ± SD: 275.8 ± 82.3 g).

### 3.2. Amniotic Cortisol and Type of Delivery

**Figure 1:**
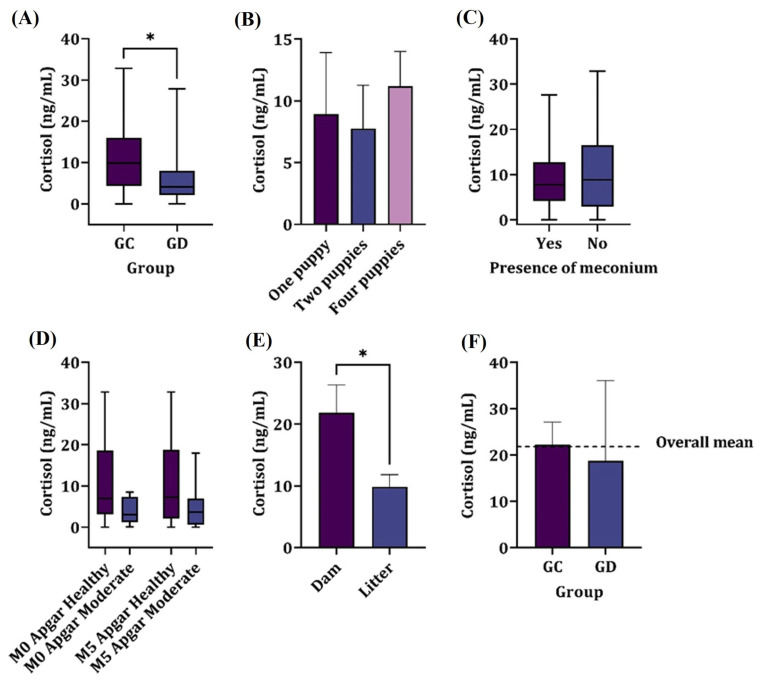
Mean cortisol concentrations in the following: (**A**) the amniotic fluid of elective c-section (GC) and therapeutic c-section (GD) groups. (**B**) The number of puppies in a litter. (**C**) The presence of meconium. (**D**) The Apgar score at M0 and M5, irrespective of the group. (**E**) The dam and litter, irrespective of the group. (**F**) The descriptive serum levels in dams. * denotes significant difference.

The cortisol levels in pups born through elective cesarean section (n = 94) showed higher levels (*p* = 0.017) compared to those from dogs undergoing therapeutic cesarean section (n = 20), as shown in Figure 1.

The median concentrations found were 9.86 ng/mL for group GC and 4.11 ng/mL for group GD. There was no significant correlation between the birth order of neonates and the obtained amniotic cortisol levels (*p* > 0.05).

### 3.3. Meconium

**Figure 2:**
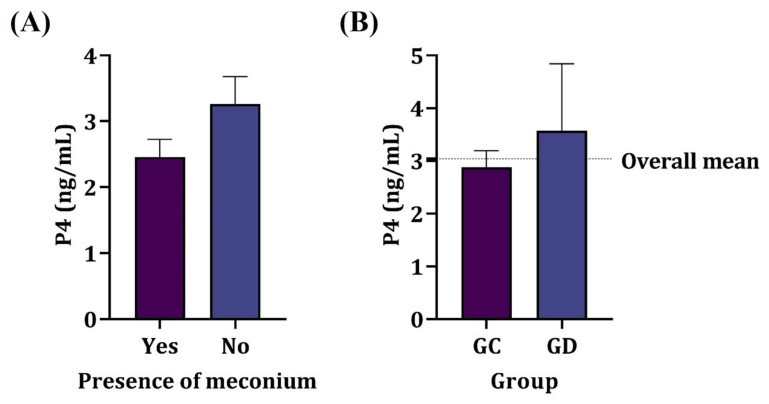
Mean serum progesterone concentration in dams. (**A**) Presence of meconium in amniotic fluid. (**B**) Elective (GC) and therapeutic (GD) cesarean section groups. No significant difference was observed (*p* > 0.05).

The presence of meconium in the amniotic fluid showed no relation to the amniotic cortisol nor P4 values of the dams (*p* > 0.05) as shown in Figure 2, regardless of the parturient condition. Most samples of the amniotic fluid containing meconium were from elective cesarean sections (87.5%); however, there was no significant association between the type of delivery and the presence of meconium in amniotic fluid (*p* > 0.05).

### 3.4. Neonatal Viability

After birth, the pups underwent resuscitation maneuvers, including cleaning and suctioning of the airways, rubbing of the chest, and slow and progressive warming. The effectiveness of these interventions was evaluated based on the changes in the Apgar score and the observable signs of vitality, such as mobility, vocalization, suckling reflex, and muscle tonus.

Table 1 summarizes the findings on neonatal viability, assessed using the Apgar scale for 44 individuals. At moment M0 (immediately after cardiopulmonary resuscitation), most neonates delivered via elective cesarean section (92.86%) showed no distress, compared to most neonates delivered via therapeutic cesarean section (56.25%) who exhibited moderate distress. A similar pattern was observed at moment M5 (5 min after cardiopulmonary resuscitation), with 89.29% of neonates in GC group showing no distress, while 56.25% of those in the GD group still showed moderate distress.

A highly significant correlation (*p* ≤ 0.001) was found between the type of delivery and the level of neonatal distress at both evaluated time points, with neonates from therapeutic cesarean section experiencing higher distress levels. However, no association was found between the degree of distress and cortisol levels in the amniotic fluid (*p* > 0.05), nor between neonatal distress and the presence of meconium in the amniotic fluid (*p* > 0.05).

### 3.5. Birth Order, Litter Size, and Neonatal Weight

**Figure 3:**
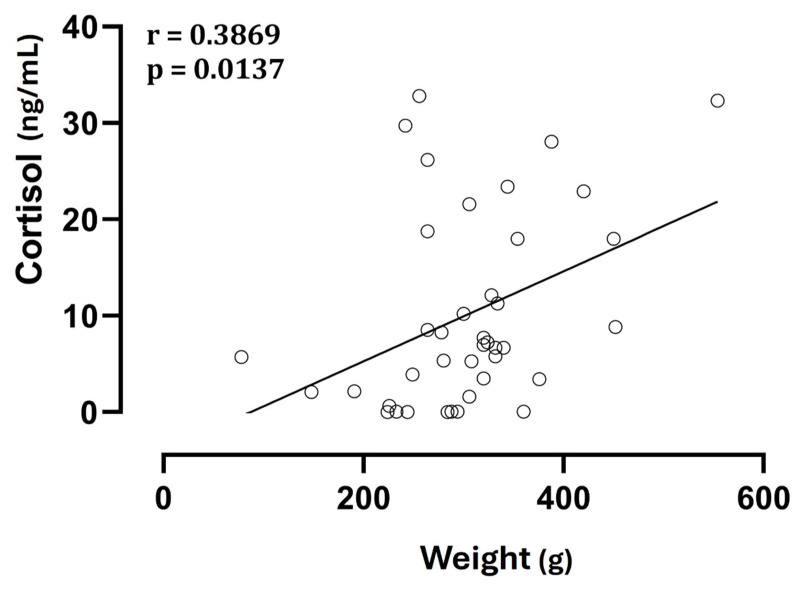
Correlation between neonatal weight and cortisol levels in amniotic fluid.

No significant differences (*p* > 0.05) were observed in cortisol concentrations among fetuses with the same birth order but from different types of delivery, and there was no correlation between litter size and cortisol levels, both fetal and maternal (*p* > 0.05). On the other hand, neonatal weight showed a direct correlation with cortisol levels (Figure 3).

### 3.6. Maternal Blood

There was no significant association between progesterone levels and the type of delivery or the presence of fetuses with meconium in the amniotic cavity (*p* > 0.05). The mean progesterone level was just below 3 ng/mL in the elective cesarean section group and exceeded 3 ng/mL in the therapeutic cesarean section group.

## 4. Discussion

This study focused on evaluating serum cortisol levels in maternal blood and amniotic fluid, considering different variables to assess fetal maturity and stress in neonates. Our results indicated higher cortisol levels in the amniotic fluid of fetuses from elective cesarean sections compared to those from therapeutic c-sections. This finding contrasted with previous reports by Bolis et al. [11] and Fusi et al. [12]. For instance, Bolis et al. [11] found an average cortisol level of 3.7 ng/mL in the amniotic fluid of canine fetuses born via elective cesarean sections, particularly in breeds predisposed to dystocia (Pug, Chihuahua, Bull Terrier, German Spitz, Maltese, and Dachshund). Notably, they observed that puppies that did not survive beyond 24 h had higher cortisol levels than those that did.

Similar cortisol concentrations were noted by Plavec et al. [13] in elective cesarean deliveries, whereas significantly higher levels (14.01 ± 1.10 ng/mL) were recorded in therapeutic cesarean sections addressing dystocia. In our study, the mean cortisol level in elective c-section was 9.86 ng/mL, compared to 4.11 ng/mL in therapeutic c-section. This finding was particularly intriguing, as dystocia, which is an acute stress condition, would typically be expected to result in elevated hormone levels. The apparent contradiction may be multifactorial, necessitating a comprehensive analysis of the circumstances surrounding each type of cesarean procedure, as well as variations in breed, the type of dystocia, the duration of dystocia, and the sample size of the study groups.

In a related study analyzing cortisol concentrations in the blood of bitches and their neonates during the perinatal period, it was observed that maternal dystocia resulted in elevated cortisol levels in the bitches immediately after delivery [14]. Conversely, only fetal dystocia was associated with increased cortisol concentrations in the neonates, indicating differences in maternal and neonatal hormonal responses depending on the type of dystocia. In our study, the bitches experienced dystocia for up to 6 h, all exhibiting uterine inertia, which could have influenced the results as progesterone plays a significant role in the pathophysiology of primary uterine inertia in bitches [15]. Additionally, it is important to consider that our evaluation of cortisol was based on amniotic fluid, which has a complex composition, potentially influenced by contributions from maternal, placental, and fetal physiological compartments [12].

A reduction in progesterone is necessary for the initiation of labor; therefore, in cases of dystocia where labor fails to initiate, elevated progesterone levels may correlate with low fetal cortisol levels, which typically rise to facilitate labor. Studies have indicated that the maturation of the fetal pituitary–adrenal axis is critical for this process [6,16]. Consequently, if the female does not enter active labor and progesterone remains elevated, fetal cortisol levels may not rise as expected, leading to a reduced stress response during cesarean section. Accordingly, the literature recommends that elective cesarean sections should be performed only after progesterone levels fall below 2 ng/mL, which is considered an indicator of fetal maturity [17,18]. In our study, progesterone levels in the dystocia group were above 3 ng/mL, while those in the elective group were consistently below this threshold. Although the progesterone level in neither group was considered ideal for performing a c-section, ultrasound evaluation was utilized as a reference to determine the optimal timing for intervention and to minimize fetal depression. Importantly, the timing of intervention was validated, as most of the neonates exhibited no distress on the Apgar score evaluations. These findings underscore the necessity of conducting a comprehensive obstetrical assessment that considers various parameters to inform the best timing for intervention. The variability in physiological responses across different breeds and individuals may significantly influence neonatal outcomes.

In terms of neonatal viability, our findings revealed that approximately 56.25% of the fetuses delivered via therapeutic cesarean sections exhibited Apgar scores indicating moderate distress, whereas 92.86% of those born through elective cesarean sections showed no signs of distress. While we did not find a significant association between the degree of distress and cortisol levels, the notable correlation between the type of delivery and neonatal distress highlighted the increased risk of adverse outcomes for fetuses delivered through therapeutic cesarean sections. This aligned with previous research linking Apgar scores to neonatal viability, particularly in brachycephalic breeds, which are more susceptible to dystocia and its consequences, including higher neonatal mortality rates [19].

The presence of meconium in the amniotic fluid is widely recognized as an indicator of fetal stress related to hypoxia in humans, associated with higher rates of emergency cesarean sections, the need for neonatal resuscitation, alterations in cardiotocography exams, and lower Apgar scores [20,21]. However, meconium can also be observed in uncomplicated deliveries, where neonates display adequate vitality, largely depending on the quantity and viscosity of the meconium [19,22]. In our study, the absence of a significant relationship between the presence of meconium and fetal cortisol values suggested that its passage is more associated with the maturation process than with the degree of fetal distress [23]. Furthermore, the fact that 87.5% of the meconium samples were from the elective c-section group supported the assumption that the procedures were performed at the appropriate time, considering fetal maturation. This was further validated by the lack of association between the type of delivery and the degree of neonatal distress in cases involving meconium [24].

Nonetheless, when meconium is present in larger quantities or has a thick consistency, it is linked to more severe neonatal outcomes, such as meconium aspiration syndrome (MAS), which is one of the leading causes of perinatal death. MAS can result in respiratory complications, ranging from mild issues to persistent pulmonary hypertension of the newborn (PPHN), even with adequate ventilatory support [25]. These potential complications underscore the importance of careful ultrasound monitoring during late pregnancy in dogs, particularly in cases of elective c-section, to enable appropriate interventions when large quantities of meconium are present in the amniotic fluid. Additionally, other factors may influence neonatal outcomes [26,27]. Given the scarcity of studies on this topic, ongoing research into the relationship between meconium, neonatal health, and effective interventions is essential for reducing mortality and improving the vitality of newborn dogs [28,29].

Studies have shown that both large and very small litters are associated with reduced puppy survival at birth [30,31]. On the other hand, a study with brachycephalic dogs showed higher neonatal viability in puppies born in larger litters compared to those born in smaller ones [32], suggesting that while larger litters may pose challenges like resource competition, they do not inherently increase maternal or fetal stress as measured by cortisol. In fact, we found no correlation between litter size and cortisol levels, neither for the neonates nor the dams, which might point out the complexity of the interactions between maternal physiology, fetal development, and environmental stressors in dogs.

Although cortisol is a widely recognized biomarker for stress, it may not fully reflect the well-being of neonates or dams. Evidence suggests that, while cortisol levels are indicative of acute stress responses, they fail to capture other critical aspects of neonatal health, such as behavioral adaptation, immune function, and developmental outcomes [33]. Future studies should include behavioral indicators, heart rate variability, immune status, and long-term development, especially when comparing different peripartum conditions like dystocia or elective cesarean sections. Integrating these markers would greatly enhance the understanding of stress and well-being in canine perinatal care and support more comprehensive physiological and clinical evaluations.

The success of cesarean section crucially depends on the timing of the procedure, both in elective approaches and therapeutic cesarean sections. In breeds and individuals with a predisposition or history of dystocia, elective cesarean sections tend to have a more favorable outcome for both the mother and the puppies, with emergency cesarean sections associated with a higher risk of stillbirths [34]. Identifying the optimal moment for intervention necessitates the meticulous monitoring and documentation of variables that indicate fetal maturity, fetal distress, and labor signaling. Such careful assessment is essential for achieving better outcomes for both the dam and her neonates.

One notable limitation of this study was the imbalance in the number of dams between the groups, with twenty-two animals in the control group (CG) and only eight in the dystocia group (DG). This disparity could potentially affect the statistical power and the robustness of the comparisons. However, it is important to note that this is a clinical study, which inherently depends on casuistic case selection. For the dystocia group, we applied very strict inclusion criteria to select only cases with similar clinical and anatomical characteristics, aiming to minimize confounding factors. This strict selection led to the exclusion of many other cases of dystocia that did not meet these criteria, but it also resulted in a more homogeneous group, which could mitigate some of the statistical limitations caused by the smaller sample size. Nonetheless, the small number of cases and the unbalanced groups were inherent limitations, and larger, prospective studies are warranted to validate and expand upon these findings. Future research exploring longer durations of dystocia and more diverse cases could provide additional insights and strengthen the evidence base.

## 5. Conclusions

Our findings demonstrated that elective cesarean sections resulted in higher cortisol levels in amniotic fluid compared to therapeutic cesarean sections, challenging previous research while emphasizing the complexity of the physiological responses involved. Notably, we observed a significant correlation between the type of delivery and neonatal distress, underscoring the increased risk of adverse outcomes associated with therapeutic cesarean sections.

Our analysis also revealed no significant relationship between the presence of meconium in the amniotic fluid and fetal cortisol levels, suggesting that meconium passage is more related to fetal maturation than to fetal distress. Additionally, our study found no correlation between the litter size and cortisol levels, indicating that environmental stressors and maternal physiology play crucial roles in neonatal health outcomes.

Overall, thorough obstetrical evaluation and monitoring are essential for making informed decisions regarding the timing of interventions, taking into account the variability in physiological responses across different breeds and individuals.

## Figures and Tables

**Table 1:** Distribution of the number (and percentage) of neonates born through elective cesarean section (GC) and therapeutic cesarean section (GD), according to the degree of distress obtained from the Apgar scale at the two moments evaluated in this study.

Experimental Group	Severe Distress (0–4)	Moderate Distress (5–9)	Absence of Distress (10–14)
Immediately after CPR *			
**GC**	1 (3.6)	1 (3.6) ^a^	26 (92.9) ^a^
**GD**	0 (0)	9 (56.3) ^b^	7 (43.8) ^b^
**5 min after CPR**			
**GC**	0 (0)	3 (10.7) ^a^	25 (89.3) ^a^
**GD**	0 (0)	9 (56.3) ^b^	7 (43.8) ^b^

* Cardiopulmonary resuscitation. Different lower-case letters (a or b) in the same column represent statistical differences in the comparison of means (*p* > 0.05).

## Data Availability

The original contributions presented in this study are included in the article. Further inquiries can be directed to the corresponding author(s).
